# Status of Institutional Review Board Meetings Conducted Through Web Conference Systems in Japanese National University Hospitals During the COVID-19 Pandemic: Questionnaire Study

**DOI:** 10.2196/22302

**Published:** 2020-11-19

**Authors:** Kenta Yagi, Kazuki Maeda, Satoshi Sakaguchi, Masayuki Chuma, Yasutaka Sato, Chikako Kane, Akiyo Akaishi, Keisuke Ishizawa, Hiroaki Yanagawa

**Affiliations:** 1 Clinical Research Center for Developmental Therapeutics Tokushima University Hospital Tokushima Japan; 2 Department of Clinical Pharmacology and Therapeutics Tokushima University Graduate School of Biomedical Sciences Kuramoto-cho, Tokushima Japan; 3 Department of Pharmacy Tokushima University Hospital Kuramoto-cho, Tokushima Japan

**Keywords:** COVID-19, IRB, Institutional Review Board, REB, Research Ethics Board, web conference, survey, drug development, teleconference, clinical trial, Japan, hospital

## Abstract

**Background:**

With the global proliferation of the novel COVID-19 disease, conventionally conducting institutional review board (IRB) meetings has become a difficult task. Amid concerns about the suspension of drug development due to delays within IRBs, it has been suggested that IRB meetings should be temporarily conducted via the internet.

**Objective:**

This study aimed to elucidate the current status of IRB meetings conducted through web conference systems.

**Methods:**

A survey on conducting IRB meetings through web conference systems was administered to Japanese national university hospitals. Respondents were in charge of operating IRB offices at different universities. This study was not a randomized controlled trial.

**Results:**

The survey was performed at 42 facilities between the end of May and early June, 2020, immediately after the state of emergency was lifted in Japan. The survey yielded a response rate of 74% (31/42). Additionally, while 68% (21/31) of facilities introduced web conference systems for IRB meetings, 13% (4/31) of the surveyed facilities postponed IRB meetings. Therefore, we conducted a further survey of 21 facilities that implemented web conference systems for IRB meetings. According to 71% (15/21) of the respondents, there was no financial burden for implementing these systems, as they were free of charge. In 90% (19/21) of the facilities, IRB meetings through web conference systems were already being conducted with personal electronic devices. Furthermore, in 48% (10/21) of facilities, a web conference system was used in conjunction with face-to-face meetings.

**Conclusions:**

Due to the COVID-19 pandemic, the number of reviews in clinical trial core hospitals has decreased. This suggests that the development of pharmaceuticals has stagnated because of COVID-19. According to 71% (15/21) of the respondents who conducted IRB meetings through web conference systems, the cost of introducing such meetings was US $0, showing a negligible financial burden. Moreover, it was shown that online deliberations could be carried out in the same manner as face-to-face meetings, as 86% (18/21) of facilities stated that the number of comments made by board members did not change. To improve the quality of IRB meetings conducted through web conference systems, it is necessary to further examine camera use and the content displayed on members’ screens during meetings. Further examination of all members who use web conference systems is required. Our measures for addressing the requests and problems identified in our study could potentially be considered protocols for future IRB meetings, when the COVID-19 pandemic has passed and face-to-face meetings are possible again. This study also highlights the importance of developing web conference systems for IRB meetings to respond to future unforeseen pandemics.

## Introduction

In 2020, the novel COVID-19 disease became a pandemic [1], causing several changes to people’s lifestyles [2]. In Japan, many individuals were infected, prompting the declaration of a state of emergency [3] for the entire country. As a result, people have avoided the “3 Cs”—closed spaces, crowds, and contact. Organizations, including institutional review boards (IRBs), have also adopted this practice, making it difficult to conduct regular face-to-face meetings. Without hospital IRBs, clinical trials cannot be conducted [4,5]. Therefore, concerns about the stagnation of drug development have been raised. Japanese ordinances indicate that IRBs should operate in all medical research institutes [5]. Within this context, the Ministry of Health, Labor, and Welfare [6] issued a notice titled “On handling of reviews by the Institutional Review Board regarding clinical trials related to the novel coronavirus,” which advised that IRB meetings were allowed to be conducted via the internet. Due to the declaration of a state of emergency and university regulations, it has become difficult to conduct face-to-face IRB meetings in a group setting at Tokushima University hospital. Therefore, we attempted to conduct IRB meetings through a web conference system at our hospital, starting on May, 2020.

Thanks to advancements in science and technology, online communication has become normal in human life due to the widespread use of the internet [7,8]. As a result, web conference systems are being adopted in places of work, rapidly gaining popularity, and expanding their market scale. Due to the COVID-19 pandemic, the demand for web communication has rapidly increased [9,10]. Web conferencing systems are also being used more in health care [11-15]. However, online communication requires an operating environment with internet connectivity and compatible devices, which can be costly. Moreover, internet meetings are relatively new, and various problems during their implementation have been reported [16-18]. Unfortunately, there are no clear, useful countermeasures for these issues. There may also be problems within IRBs, such as proper deliberation upon holding a web-based IRB meeting and the possibility of information leakage. The actual situation regarding web-based IRB meetings remains unclear because the problems surrounding such meetings have yet to be thoroughly examined. As a result, the necessary steps for conducting a successful web-based IRB meeting remains unclear. In addition, since the social environment on the internet and the laws and regulations on IRBs differ across countries, it is expected that individual, country-specific countermeasures are required. Based on the web conference system already implemented in our hospital for IRB meetings, we surveyed external members who wanted to continue having meetings through the web conference system.

Therefore, conducting IRB meetings through web conference systems may continue, even after the COVID-19 pandemic resolves. However, it is necessary to ensure the efficiency of such meetings while maintaining proper deliberations. As such, we aimed to clarify the current status of IRB meetings conducted through web conference systems and elucidate problems related to such meetings across Japan.

## Methods

### Questionnaire Administered to Japanese National University Hospitals

With the cooperation of Topic Group 1, a subcommittee of the National University Hospital Clinical Research Promotion Initiative, we administered a closed survey to participating facilities regarding IRB meetings conducted through web conference systems. The group is composed of national university hospitals. The respondents were in charge of operating the IRB office of each university, and agreements were based on the questionnaire’s answers. To understand the situation during the declaration of a state of emergency (ie, April to May, 2020), the survey was conducted immediately after its announcement (ie, end of May to early June, 2020) in Japan. The questionnaire used for the survey was based on the Checklist for Reporting Results of Internet E-Surveys (CHERRIES) checklist [19] and made using Microsoft Forms. We delivered the survey via email, and the respondents answered using a computer. To avoid largely varying opinions, the respondents were asked to select answers from predefined options. The questionnaire consisted of 39 questions and 3 pages. The third and subsequent questions were provided in a table, and they targeted facilities that had conducted IRB meetings through a web conference system. The questions are listed in Table S1 in Multimedia Appendix 1. We confirmed that there were no multiple responses from the same respondents, as there was a form for noting respondents’ university affiliations. The response rate was 74% (31/42). Although a structure was in place for contacting respondents when answers were incomplete, it was not used because no respondents were excluded from the analysis due to missing data. This survey did not require a review by the IRB because there were no items regarding personal information and this study was not a randomized controlled trial. No incentives were generated for participating in this survey.

### Analysis Methods

Based on the number of people who contracted COVID-19, we established endemic and nonendemic areas. As of May 31, 2020, a COVID-19 epidemic area is defined as a region with ≥500 COVID-19–positive cases. Additionally, the data regarding the COVID-19 epidemic were tallied in the period following Japan’s first confirmed COVID-19 case in February, 2020. With regard to items related to cost, currencies were converted from yen and stated as US dollars (¥100=US $1). In this study, external members were defined as members who were not Good Clinical Practice experts and members who were not otherwise affiliated with the involved parties.

In Japan, core hospitals are designated as sites that lead clinical trials. Since IRB operation methods and sizes may be different for core hospitals that lead clinical trials for other facilities, we collected data on the IRB environment of other facilities separately. In addition, although the participation of external committee members is required to hold an IRB meeting, the “3Cs” must be avoided for IRB participation. Therefore, the number of external committee members may be a factor in conducting IRB meetings through web conference systems. As such, data on external committee members were also separately collected.

### Statistical Analyses

**Figure 1:**
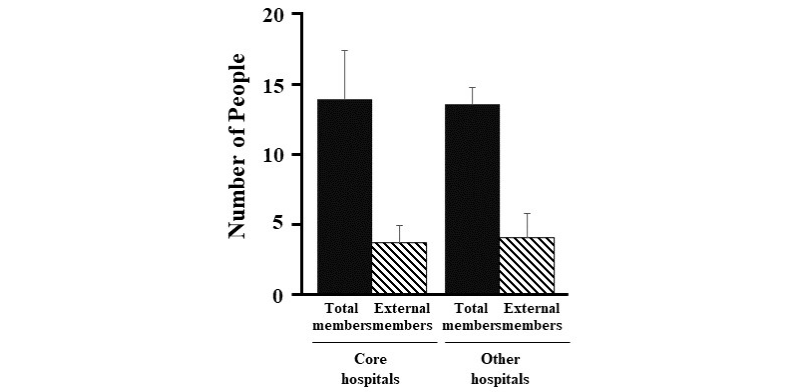
Composition of institutional review board members in Japanese university hospitals.

**Figure 2:**
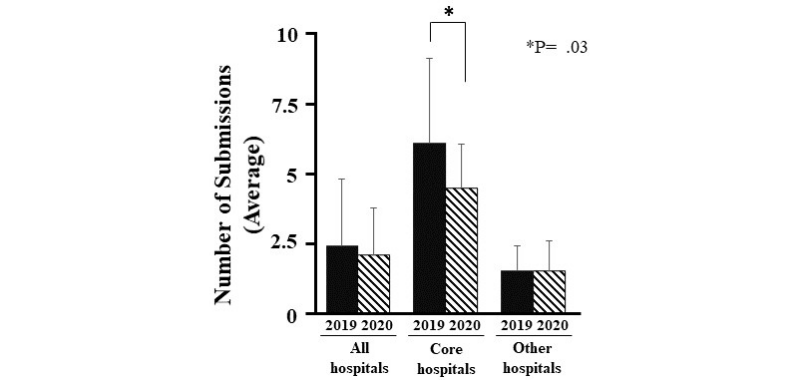
Comparison between the number of new registrations to institutional review boards at university hospitals in Japan before and after the COVID-19 pandemic.

**Figure 3:**
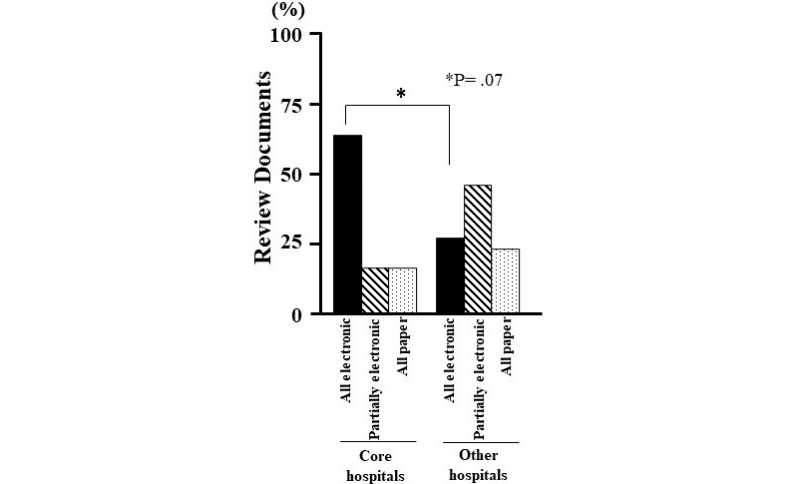
Digitization of review documents in Japanese university hospitals.

The composition of IRB members in Japanese university hospitals (Figure 1) and the number of new registrations to IRBs in Japanese university hospitals before and during the COVID-19 pandemic (Figure 2) were analyzed using the Student *t* test. The digitization of review documents in Japanese university hospitals (Figure 3) were compared using the Chi-square test. Data were shown as means with standard deviations or n (%). A *P* value of <.05 was considered statistically significant for all analyses.

## Results

### Status of Facilities Before the COVID-19 Pandemic

Of the 31 facilities that responded to the survey, 6 (19%) were clinical trial core hospitals [20]. IRB meetings were held monthly at all facilities. In addition, there was no difference in the total number of IRB members and external members based on whether the facility was a clinical research core hospital (Figure 1).

For core hospitals, all review materials were electronic in 67% (4/6) of facilities, partially electronic in 17% (1/6) of facilities, and all on paper in 17% (1/6) of facilities. For other hospitals, all review materials were electronic in 28% (7/25) of facilities, partially electronic in 48% (12/25) of facilities, and all on paper in 24% (6/25) of facilities. There was no significant difference in the number of facilities that digitized all review materials between core hospitals and other facilities (*P*=.07).

**Figure 4:**
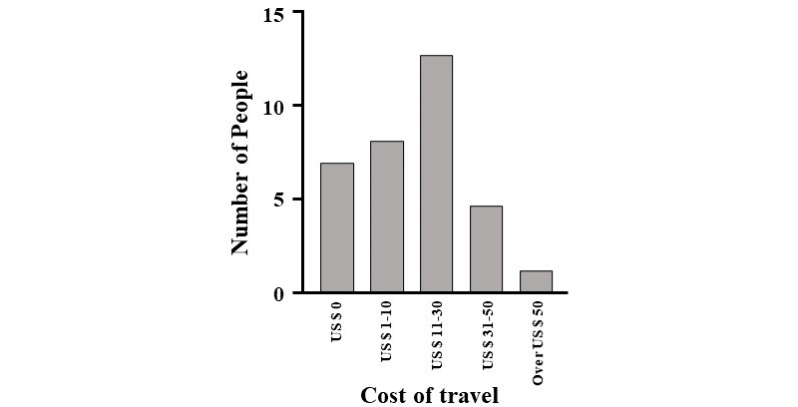
Cost required for external committee members to get to institutional review board meeting sites at university hospitals in Japan.

With regard to participation in IRB meetings, external members spent a median of US $20 per 40 minutes. It was also revealed that 16% (5/31) of respondents participated in meetings for 60 minutes or longer (Figures 2 and 4).

### Strategies or Practices During the COVID-19 Pandemic

In the Strategies or Practices During COVID-19 section of the questionnaire, we examined the relationship between the COVID-19 pandemic and the introduction of web-based IRB meetings (Table 1). Of the 31 IRB offices, 11 (35%) worked remotely due to COVID-19, while 4 (13%) opted to postpone IRB meetings. About 68% (21/31) of the facilities introduced web conference systems for IRB meetings. However, 13% (4/31) of the universities could not hold IRB meetings due to reasons unrelated to the COVID-19 pandemic and 20% (1/5) were unable to do so due to being located in a COVID-19 epidemic area. The rate of introducing web conference systems for IRB meetings was 60% (3/5) for facilities located in a COVID-19 epidemic area.

Web conference systems were also used in 81% (17/21) of facilities that introduced web-based IRB meetings (Table 1). The rate of introducing web conference systems for IRB meetings was 68% (21/31) for all hospitals, 71% (17/24) for facilities that digitized review materials, and 57% (4/7) for facilities that did not.

**Table 1:** Activity and preparation regarding web-based institutional review board meetings in university hospitals during the COVID-19 pandemic.

Activity		IRB^a^ member, n (%)
**IRB office is conducting remote work**		
	Yes	11/31 (35)
	No	20/31 (65)
**IRB meetings are cancelled or suspended**		
	Yes	4/31 (13)
	No	27/31 (87)
**IRB meetings are conducted using web conferencing tools**		
	Yes	21/31 (68)
	No	10/31 (32)

^a^IRB: institutional review board.

A further investigation was conducted on the implementation of web conference systems in facilities that held web-based IRB meetings (Table 2). Of the 21 facilities that conducted IRB meetings through web conference systems, 15 (71%) stated that the introduction cost was US $0, 19 (90%) held meetings using personal electronic devices, and all facilities kept recordings of these IRB meetings as minutes. However, the most popular web conference system, WebEX, was used in 38% (8/21) of facilities, and security measures that were implemented varied across facilities.

We also questioned whether the facilities that adopted web conference systems for IRB meetings were able to deliberate properly (Table 3). Of the 21 facilities, 15 (71%) stated that cameras were used during IRB meetings, and of those 15, 10 (67%) conducted IRB meetings with a combination of web conference systems and face-to-face meetings. The display on members’ computer screens during deliberation also varied according to the facility (ie, faces of other members, review materials, etc). Moreover, compared to in-person IRB meetings conducted before the COVID-19 pandemic, the discussion time for IRB meetings conducted with web conference systems during the COVID-19 pandemic decreased in 38% (8/21) of facilities, but the number of member comments decreased in 10% (2/21) of the facilities.

**Table 2:** Practical aspects regarding the management of institutional review board meetings conducted through web conference systems.

IRB^a^ meeting characteristics		IRB member, n (%)	
**Cost of hosting an IRB meeting using a web conference system (US $)**			
	0		15/21 (71)
	100-500		2/21 (10)
	501-1000		1/21 (5)
	Unknown		3/21 (14)
**Confirmation of attendance**			
	Using the list of participants in the web conferencing system		2/21(10)
	Confirmation of attendance through live attendance		11/21 (52)
	Voice confirmation		7/21(33)
	Checklist of participants attending the web conference and confirmation through live attendance		1/21 (5)
**Ownership of devices used**			
	Own devices		19/21 (90)
	Provided by the committee, if needed		1/21 (5)
	Distributed by the committee		1/21 (5)
**Information for keeping track of IRB meetings^b^**			
	Note in the logbooks that the meeting was held on the web		21/21 (100)
	Description of the system in use		5/21 (24)
	Note on security policy		3/21 (14)
	Note that there was enough time for discussion		3/21 (14)
	Location of each committee member's place of participation		1/21 (5)
**Web conference system used for IRB meeting**			
	Google Meet		2/21 (10)
	Skype		2/21 (10)
	Teams		2/21 (10)
	WebEX		8/21 (38)
	Zoom		7/21 (33)
**Security policy**			
	Requested to be considerate of the surrounding environment during committee meetings		11/21 (52)
	Use of the campus network		6/21 (29)
	Installation of security software		6/21 (29)
	Video recording is not allowed.		2/21(10)
	Warning about the handling of the URLs distributed for conference participation		1/21 (5)
	Entering a password to join a web conference		2/21(10)
	Management and tracking of participant accounts		3/21 (14)
	Updated operating system software		1/21 (5)

^a^IRB: institutional review board.

^b^Multiple options could be selected.

**Table 3:** Influence of the introduction of web conference systems for institutional review board meetings on the review process.

Activity		IRB^a^ member, n (%)	
**Using video in web-based IRB meeting**			
	All members		15/21 (71)
	According to member’s choice		6/21 (29)
**Members' information displayed on screen during the meeting**			
	Committee members on screen through camera		6/21 (29)
	Review materials and participating committee members		14/21 (66)
	Participant's choice		1/21 (5)
**Members Using the Web Conferencing System**			
	All members		11/21 (52)
	External members only		3/21 (14)
	Participant's choice		7/21 (33)
**Confirmation of decisions made**			
	Using the tools provided by the web conference system		1/21 (5)
	All committee members are involved prior to decision making		1/21 (5)
	Raising of hands		1/21 (5)
	Verbal confirmation		17/21 (81)
	Making use of the review table		1/21 (5)

^a^IRB: institutional review board.

### Comparison of IRB Meetings Conducted Before and During the COVID-19 Pandemic

The number of new clinical trial reviews before and during the COVID-19 pandemic (ie, February to May, 2019 and February to May, 2020, respectively) was analyzed to investigate the impact of the COVID-19 pandemic on the conduction of clinical trials. Following the onset of the pandemic, the number of reviews conducted at clinical trial core hospitals decreased (Figure 2). No facility shortened the discussion time by more than 30 minutes, and the number of comments from committee members remained consistent in 86% (18/21) of the facilities (Table 4).

**Table 4:** Comparison of the discussion characteristics of institutional review board meetings conducted through web conference systems during the COVID-19 epidemic and those of in-person meetings conducted before the COVID-19 epidemic.

Discussion characteristics		IRB^a^ member, n (%)	
**Discussion length compared to in-person meetings**			
	Shorter by <30 minutes		8/21 (38)
	Longer by <30 minutes		2/21(10)
	Longer by >30 minutes		1/21 (5)
	Same as before		10/21 (48)
**Number of comments made by committee members compared to in-person meetings**			
	More than 1.5 times		1/21 (5)
	Less than half		2/21(10)
	Same as before		18/21 (86)

^a^IRB: institutional review board.

## Discussion

### Principal Results

This study clarifies the problems that need to be solved to improve IRB meetings conducted through web conference systems. This was done by collecting information on the implementation of web conference systems for IRB meetings in Japan's national university hospitals.

In Japan, the declaration of a state of emergency [3] prompted the need to reexamine the way IRB meetings should be held. Along with this, the Ministry of Health, Labor, and Welfare issued a notice [5], tentatively allowing IRB meetings to be held in a nonassembly form. In this study, we carried out a questionnaire survey among national university hospitals in the country after the official notice to understand the problems concerning IRB meetings conducted through web conference systems. In addition, we have identified the problems that need to be addressed to improve the quality of such IRB meetings.

After the onset of the COVID-19 pandemic, the number of reviews in clinical trial core hospitals has decreased compared to those before the pandemic. However, there were no significant changes in the number of reviews in other hospitals (Figure 2). This may have been influenced by the fact that 50% (3/6) of the clinical trial core hospitals were located in COVID-19 hotspots and that the number of applications per month at other hospitals was already low (ie, 1.5 applications on average) before the pandemic. However, since the number of reviews has decreased in clinical trial core hospitals, which oversee several clinical trials, there is growing concern about the stagnation of pharmaceutical development due to COVID-19. Therefore, it is important to advance developments in IRB meetings conducted through web conference systems to prepare for future unforeseen pandemics that follow the COVID-19 pandemic.

**Figure 5:**
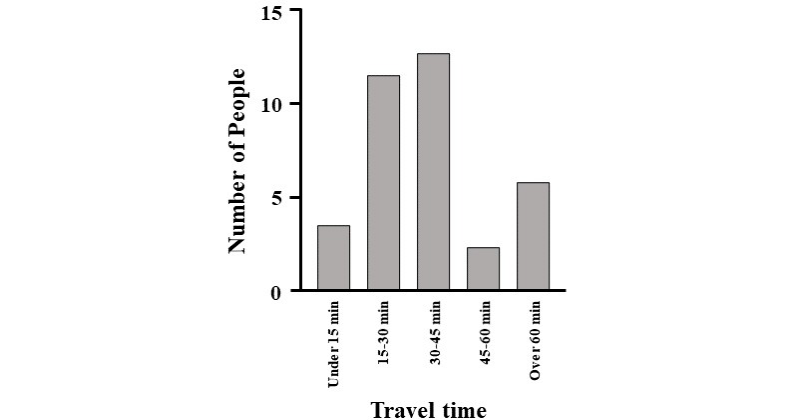
Time required for external committee members to get to institutional review board meeting sites at university hospitals in Japan.

Conducting IRB meetings through web conference systems allows people to avoid overcrowding, prevent infection, participate regardless of location, and reduce the time and cost required to commute to IRB meeting venues (Figures 4 and 5). Therefore, continuing the implementation web conference systems for IRB meetings may increase the number of attendees. Moreover, in the current IRB format, ensuring the quality of external members is difficult, especially in provincial cities, as these members would have to commute from neighboring areas. If the quality of IRBs can be guaranteed, even when using a web conference system, more advanced discussions will be possible. Web conference systems allow for the easy invitation of people who have deep insights, but are unable to join regular meetings due to the distance of IRB meeting venues.

Based on our survey on the implementation of web conference systems for IRB meetings, we were able to clarify certain aspects related to such meetings. For instance, there was no significant change in facilities that delayed IRB meetings, even in facilities located in a COVID-19 endemic area. There was also no significant increase in the rates of implementing web conference systems for IRB meetings at facilities located in a COVID-19 endemic area. Furthermore, the introduction rate of web conference systems was high in facilities that digitized their review materials (Figure 3). The digitization of review materials may have influenced the introduction of web conference systems for IRB meetings. In addition, 71% (15/21) of the facilities spent US $0 in the introduction of such meetings (Table 1). This indicated that facilities that did not adopt a web conference system would have incurred almost no financial cost in implementing a web conference system. This may be because 90% (19/21) of the facilities held meetings using personal devices. In addition, internet connectivity was identified as the most common issue in the introduction of web conference systems. However, this issue can be resolved in the future with advancements in science and technology [7,8]. Our results suggest that financial cost is not an obstacle in the introduction of web conference systems for IRB meetings. However, since such meetings rely on an internet connection, it is necessary to carry out measures for unanticipated issues that are nonexistent during face-to-face meetings.

In our survey, the web conference systems varied depending on the facility, and even the most used system, WebEX, was only used by 38% (8/21) of facilities. Further, if IRB information is compromised, many facilities, such as companies and universities, would suffer great losses. Security measures are important for preventing information leakage, but these measures are not standardized among facilities (Table 2). Nevertheless, all security measures implemented by the surveyed facilities were considered appropriate. In order to prevent information leakage in facilities with poor security measures, the country’s regulatory boards and organizations are expected to release guidelines concerning the improvement of implementing web conference systems, including guidelines for security measures.

Of the 21 facilities that introduced web conference systems for IRB meetings, 15 (71%) stated that they would continue to hold web-based meetings. Therefore, it is likely that such IRB meetings will continue, even after the pandemic, when assembly IRB meetings can be resumed. However, there are concerns that the use of conferencing systems will reduce the quality of IRB meeting deliberation. Our survey was conducted to clarify the measures needed to improve the quality of IRB meetings conducted through web conference systems.

This study revealed that the use of cameras during meetings, the content displayed on computer screens, and the scope of members who participate in meetings using the web conference system differed depending on the facility (Table 3). Additionally, compared to in-person IRB meetings conducted before the COVID-19 pandemic, no facility shortened the time of discussion by more than 30 minutes for web-based IRB meetings, and 86% of facilities stated that IRB members made the same number of comments during such meetings (Table 4). Based on these results, in order to improve the quality of IRB meetings conducted through web conference systems, it is necessary to further examine members’ use of cameras, the content displayed on members’ computer screens, and the scope of members who participate in meetings using a web conference system.

Due to the COVID-19 pandemic, the overall number of reviews in clinical trial core hospitals has decreased. Additionally, while 68% (21/31) of surveyed facilities introduced web conference systems for IRB meetings, 13% (4/31) postponed IRB meetings. According to the 71% (15/21) of the respondents who implemented web conference systems for IRB meetings, the cost of implementation was US $0, showing that there is almost no financial burden for implementing web conference systems. In 90% (19/21) of the facilities, web-based IRB meetings were already being conducted using personal electronic devices. Furthermore, in 48% (10/21) of the facilities, the web conference system was used in conjunction with face-to-face meetings. This study revealed that the use of cameras during meetings, the content displayed on computer screens, and the scope of members who participate in meetings using a web conference system differed depending on the facility.

Based on our results, we found that although many facilities could create a system for conducting web-based IRB meetings, the methods for implementing such meetings were not uniform among facilities. It is necessary to ensure the quality of IRB discussions, even if a web conferencing system is used. Further studies on the use of cameras, the content displayed on computer screens, and the scope of members who participate in meetings using a web conference system are necessary to ensure the quality of the system.

### Limitations

This study may have some limitations. For instance, our survey was administered to hospitals in Japan. Since the laws regarding clinical trials vary from country to country, the measures obtained in this study may not necessarily apply to other countries. In addition, since the survey was conducted under special circumstances (ie, the COVID-19 pandemic), it is necessary to carefully discuss whether our findings can be applied to situations after the COVID-19 pandemic. Some respondents stated that the attendance rate increased due to the introduction of web conference systems for IRB meetings, but it cannot be denied that this may be due to the decrease in the number of business trips and other meetings caused by the impact of COVID-19. As such, the attendance rate during web-based IRB meetings must be reviewed after the end of the pandemic.

### Conclusions

In this study, we identified the problems in conducting IRB meetings through web conference systems. It is necessary to improve the quality of such IRB meetings by investigating and verifying the measures for solving these problems. The results of this study can be used to guide future IRB meetings held after the end of the COVID-19 pandemic, once it becomes possible to hold face-to-face meetings. Our results are not limited to IRBs and can be used as a reference for introducing a web conference system to other organizations.

## Appendix

Multimedia Appendix 1 Table S1. The questions in the Questionnaire.

## Abbreviations

IRB: institutional review board
