# Supplementary material for: Status of Institutional Review Board Meetings Conducted Through Web Conference Systems in Japanese National University Hospitals During the COVID-19 Pandemic: Questionnaire Study
Source: J Med Internet Res. 2020 Nov 19;22(11):e22302. doi: 10.2196/22302 (PMC7683025; doi:10.2196/22302)
Supplement: Multimedia Appendix 1 [file jmir_v22i11e22302_app1.docx]

|  | Question | List of choices | | | | |  |  |
| --- | --- | --- | --- | --- | --- | --- | --- | --- |
| To all facilities | Name of the university | Free comments |  |  |  |  | |  |
|  | IRB has been cancelled or suspended | Yes | No |  |  |  | |  |
|  | Conducting remote work involved in the IRB office | Yes | No |  |  |  | |  |
|  | Number of new reviewers to IRB | Number |  |  |  |  | |  |
|  | Digitalization of review documents | Electronic | Partially electronic | Paper |  |  | |  |
|  | Number of IRB Members | Number |  |  |  |  | |  |
|  | Number of external IRB Members | Number |  |  |  |  | |  |
|  | Time required for external committee members to go to IRB site | Under 15 min | 15-30 min | 30-45 min | 45-60 min | 60 min plus | |  |
|  | Cost required for external committee members to go to IRB site | $0 | $ 1-10 | $11-30 | $31-50 | $50 plus | |  |
|  | Web-IRB was held using web conferencing tools | Yes | No |  |  |  | |  |
| To facilities hosting Web-IRB | Web conference system used in Web-IRB | Teams | ZOOM | Google Meet | Skype | Other | |  |
|  | Cost of hosting a Web-IRB | 0 | $ 1-100 | $101-500 | $501 - 1000 | Unknown | |  |
|  | Using Video in Web IRB Meeting | All members | User's choice | None | Other |  | |  |
|  | Members' information displayed on screen during the meeting | See committee members on screen through camera | Review materials and see participating committee members | Only review materials | Participant's choice | Other | |  |
|  | Members using the web conferencing system | All members | External members only | Participant's choice | Other |  | |  |
|  | Confirmation of attendance | Using the list of participants in the web conferencing system | Confirmation of attendance through live attendance | Voice confirmation | Check list of participants attending the web conference and confirmation through live attendance | Other | |  |
|  | Ownership of devices used | Own devices | Provided by the committee if needed | Distributed by the committee | Other |  | |  |
|  | Discussion Length Compared to In-Person Meetings | Shorter (less than 30 minutes) | Shorter (over 30 minutes) | Longer (less than 30 minutes) | Longer (over 30 minutes) | Same as before | |  |
|  | Number of comments by committee members | More than 1.5 times | Less than half | Same as before |  |  | |  |
|  | Confirmation of decisions made | Use the tools provided through the web conference system | All committee members are involved prior to decisionmaking | Raising of hands | Confirmed verbally | Use the review table | | Other |
|  | How to keep track of Web-IRB (select multiple options) | Note in the logbooks that the meeting was held on the web | Description of the system in use | Note on security policy | Note on security policy | Location of each committee member's place of participation | | Other |
|  | Security policy | Requested to be considerate of the surrounding environment during committee meetings | Use of the campus network | Installation of security software | Video recording is not allowed | Other | |  |
|  |  | Warning about the handling of the URLs distributed for conference participation | Enter password to join a web conference | Management and tracking of participant accounts | Update OS software |  | |  |
